# Supplementary material for: Combining the Peptide RWQWRWQWR and an Ethanolic Extract of Bidens pilosa Enhances the Activity against Sensitive and Resistant Candida albicans and C. auris Strains
Source: J Fungi (Basel). 2023 Aug 2;9(8):817. doi: 10.3390/jof9080817 (PMC10455339; doi:10.3390/jof9080817)
Supplement: Supplementary file 1 [file jof-09-00817-s001.zip › jof-2445637-supplementary.pdf]

Journal of *Fungi*

Supplementary Material.

Combining the Peptide RWQWRWQWR and an Ethanolic Extract of *Bidens pilosa* Enhances the Activity against Sensitive and Resistant *Candida albicans* and *C. auris* Strains

Yerly Vargas-Casanova [1], Claudia Patricia Bravo-Chaucanés [1], Andrea Ximena Hernández Martínez [2], Geison Modesti Costa [2], Jorge Luis Contreras-Herrera [3], Ricardo Fierro Medina [4], Zuly Jenny Rivera-Monroy [4], Javier Eduardo García-Castañeda [4] and Claudia Marcela Parra-Girado [1,*]

[1] Microbiology Department, Faculty of Sciences, Pontificia Universidad Javeriana, Bogotá 110231, Colombia; y.vargasc@javeriana.edu.co (Y.V.-C.); claub06@gmail.com (C.P.B.-C.)
[2] Chemistry Department, Faculty of Sciences, Pontificia Universidad Javeriana, Bogotá 110231, Colombia; hernandez_a@javeriana.edu.co (A.X.H.M.); modesticosta.g@javeriana.edu.co (G.M.C.)
[3] Faculty of Engineering and Basic Sciences, Insitituto Tecnológico del Putumayo, Mocoa 860001, Colombia; jorge.contreras@itp.edu.co
[4] Faculty of Sciences, Universidad Nacional of Colombia, Bogotá 111321, Colombia; rfierrom@unal.edu.co (R.F.M.); zjriveram@unal.edu.co (Z.J.R.-M.); jaegarciaca@unal.edu.co (J.E.G.-C.)
* Correspondence: claudia.parra@javeriana.edu.co

Table of content

Figure S1. Mass spectrum of Chlorogenic Acid (peak 1). 2

Figure S2. Mass spectrum of Rutin (peak 2) 2

Figure S3. Mass spectrum of Okanin-O-glucoside (peak 3) 3

Figure S4. Mass spectrum of 3,4-di-Caffeoylquinic acid (peak 4) 3

Figure S5. Mass spectrum of 3,5-di-Caffeoylquinic acid (peak 5) 4

Figure S6. Mass spectrum of Okanin 4′-O-(diacetyl)-glucoside (peak 6) 4

Figure S7. Mass spectrum of peak 7.......... 5

Figure S8. Mass spectrum of peak 8 5

Figure S9. Mass spectrum of Okanin 4′-O-(triacetyl)-glucoside (peak 9).......... 6

Figure S10. Mass spectrum of Okanin 4′-O-(triacetyl)-glucoside isomer (peak 10) 6

Figure S11. Mass spectrum of peak 11.......... 7

Table S1. Synergy test of *B. pilosa* extract/Peptide combinations against *C. albicans* SC5314. 7

Figure S12. Cell viability assay of *C. albicans* SC5314 after treatment (48 h) with R-1-R, *B. pilosa* extract to MIC value, sub MIC values and combinations of peptide and extract where there was a synergistic effect. 8

Figure S13. Cell viability assay of *C. albicans* 256 after treatment (48 h) with R-1-R$_I$, *B. pilosa* extract to MIC value, sub MIC values and combinations of peptide and extract where there was additive effect. 9

Figure S14. Cell viability assay of *C. auris* 435 after treatment (48 h) with R-1-3I, *B. pilosa* extract to MIC value, sub MIC values and combinations of peptide and extract where there was synergistic effect... 10

Figure S15. Cell viability assay of *C. auris* 537 after treatment (48 h) with R-1-R, *B. pilosa* extract to MIC value, sub MIC values and combinations of peptide and extract where there was synergistic effect. 11

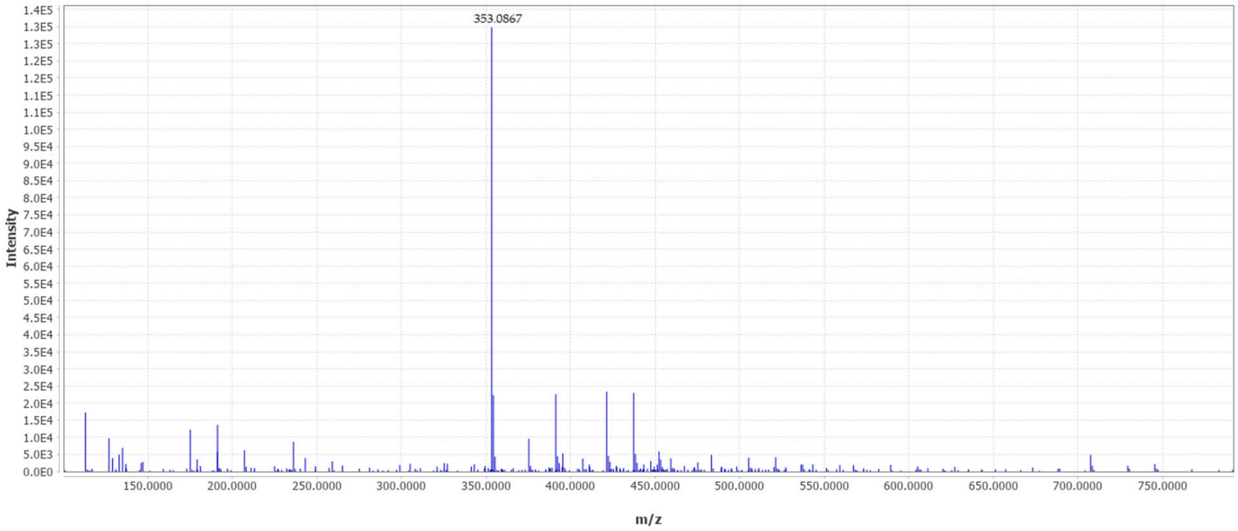

Figure S1. Mass spectrum of Chlorogenic Acid (peak 1).

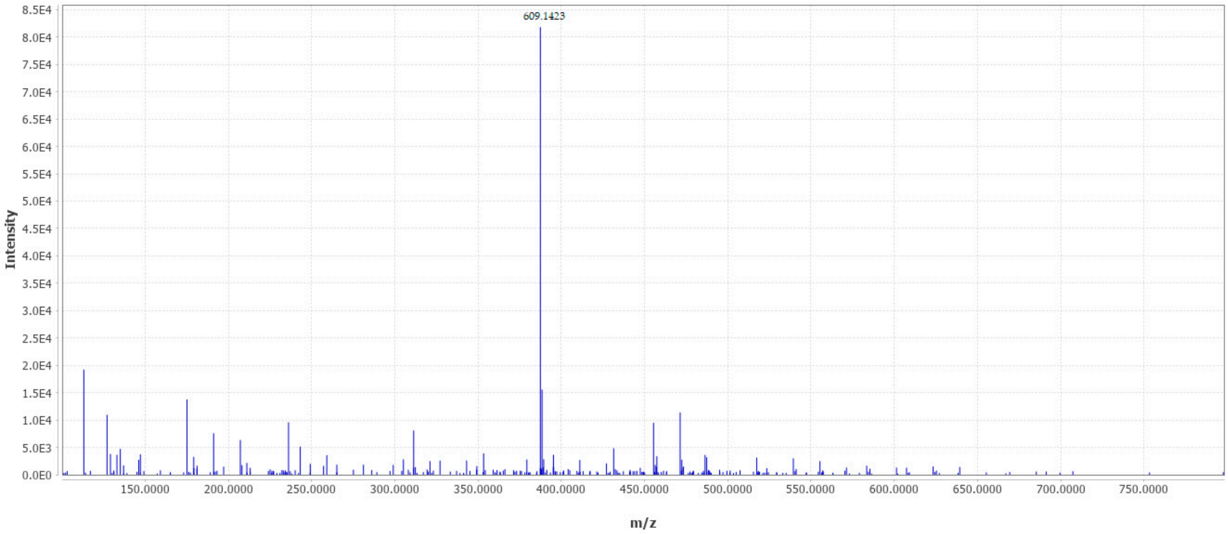

Figure S2. Mass spectrum of Rutin (peak 2)

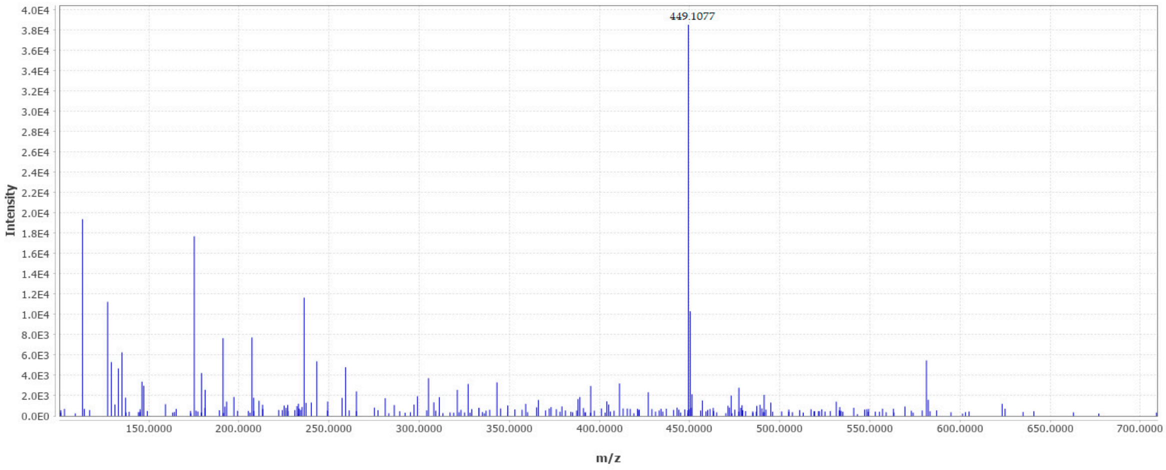

Figure S3. Mass spectrum of Okanin-O-glucoside (peak 3)

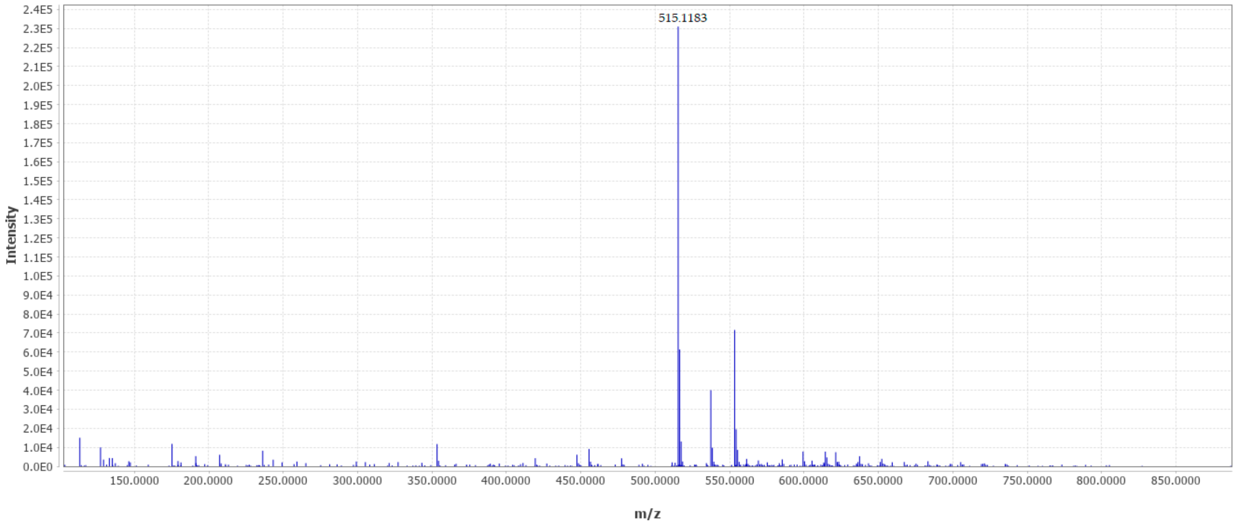

Figure S4. Mass spectrum of 3,4-di-Caffeoylquinic acid (peak 4)

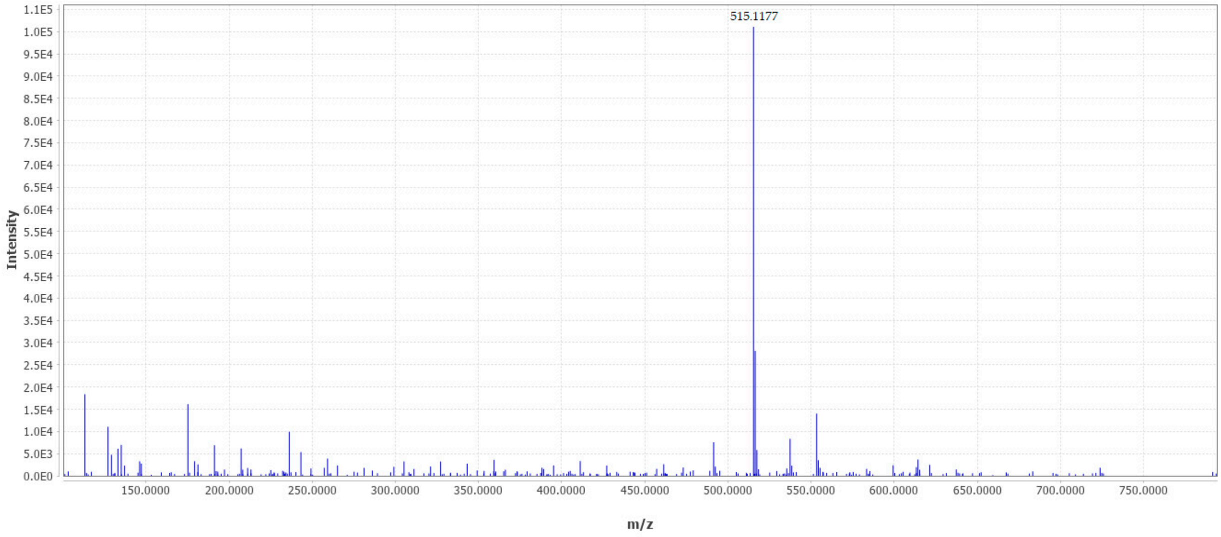

Figure S5. Mass spectrum of 3,5-di-Caffeoylquinic acid (peak 5)

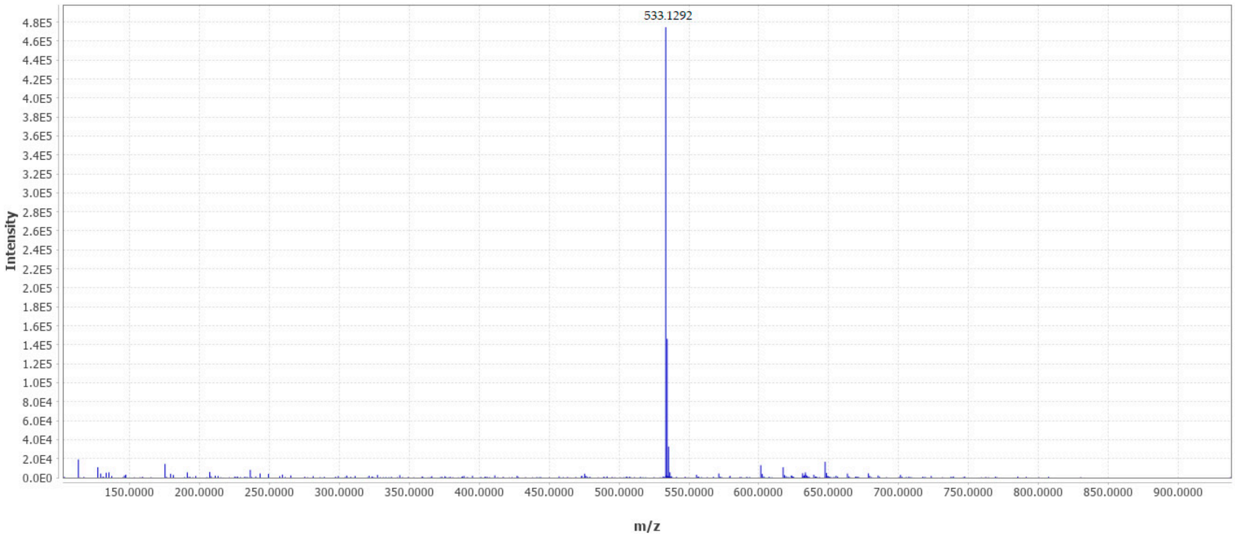

Figure S6. Mass spectrum of Okanin 4′-O-(diacetyl)-glucoside (peak 6)

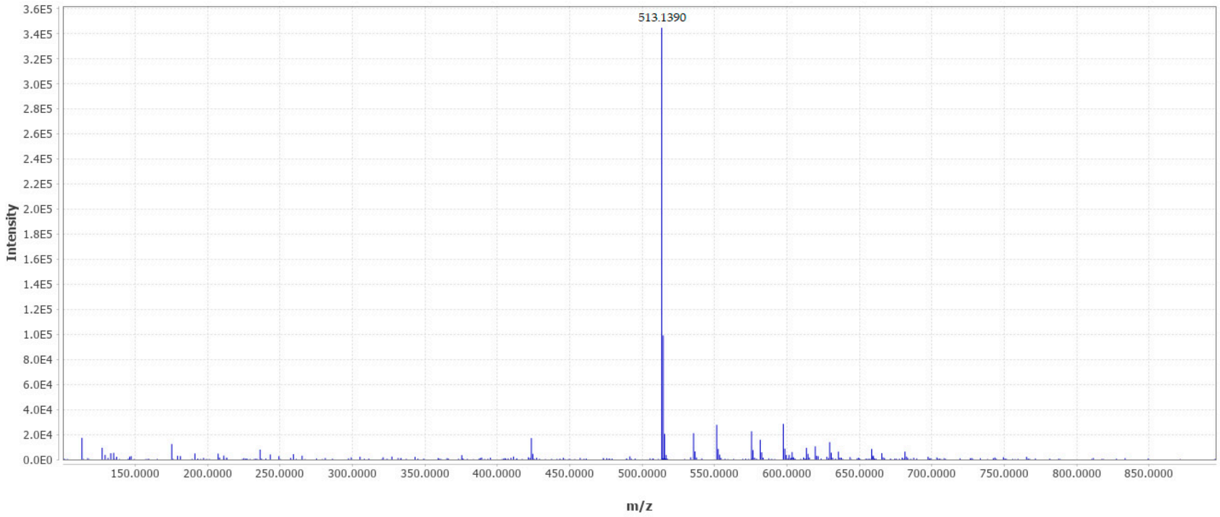

Figure S7. Mass spectrum of peak 7

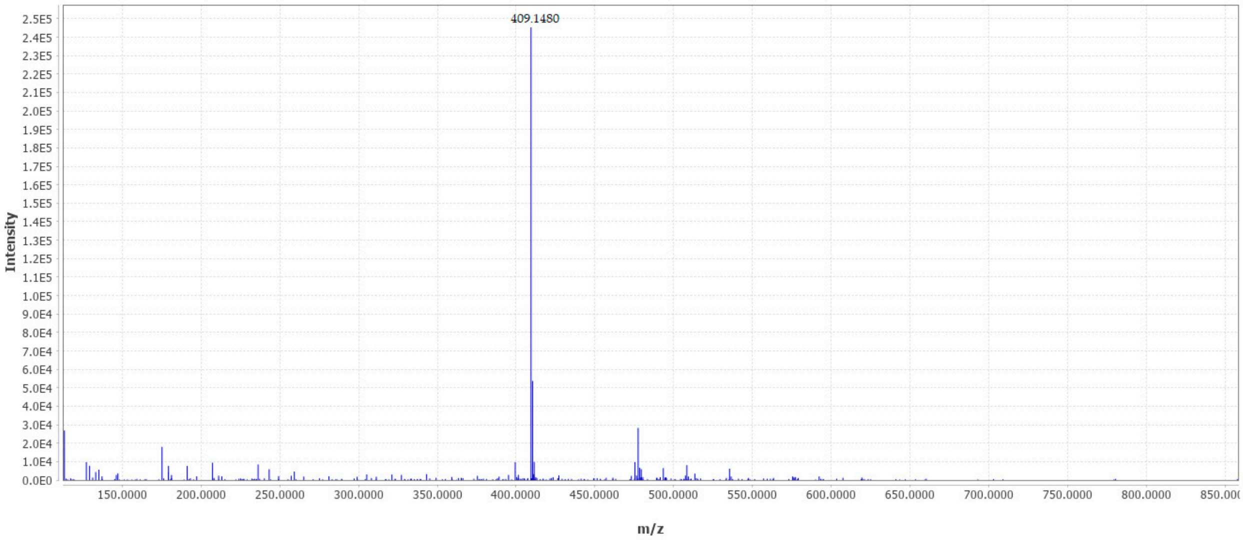

Figure S8. Mass spectrum of peak 8

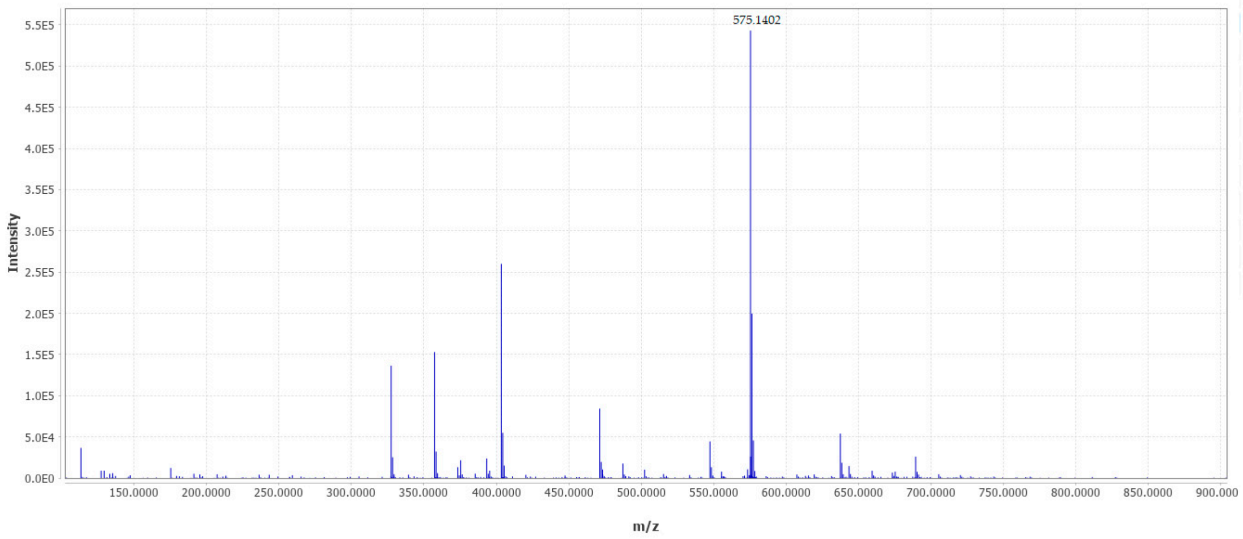

Figure S9. Mass spectrum of Okanin 4′-O-(triacetyl)-glucoside (peak 9)

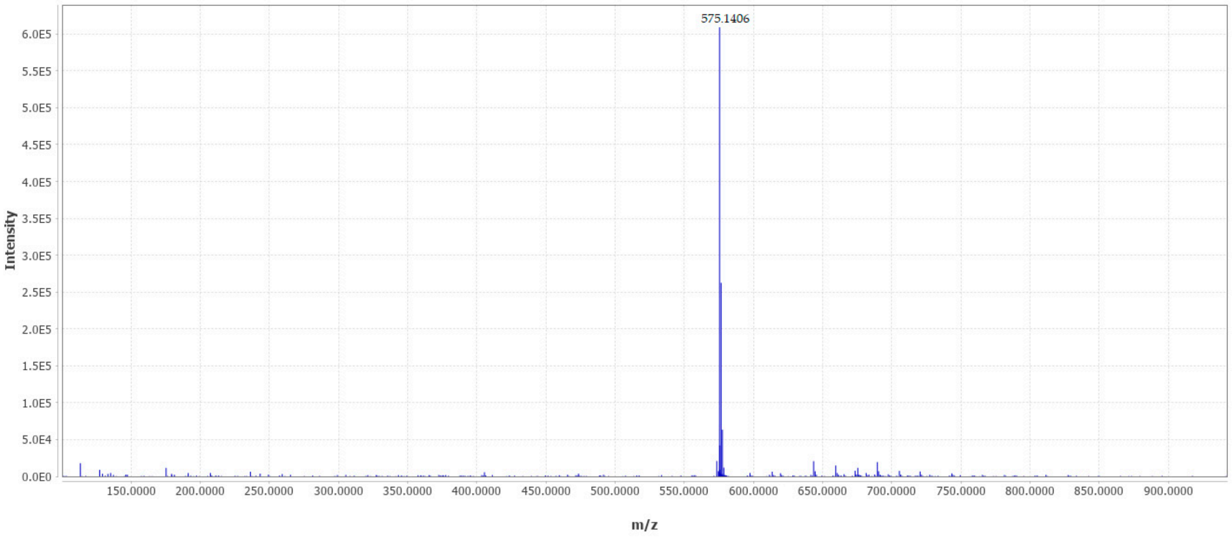

Figure S10. Mass spectrum of Okanin 4′-O-(triacetyl)-glucoside isomer (peak 10)

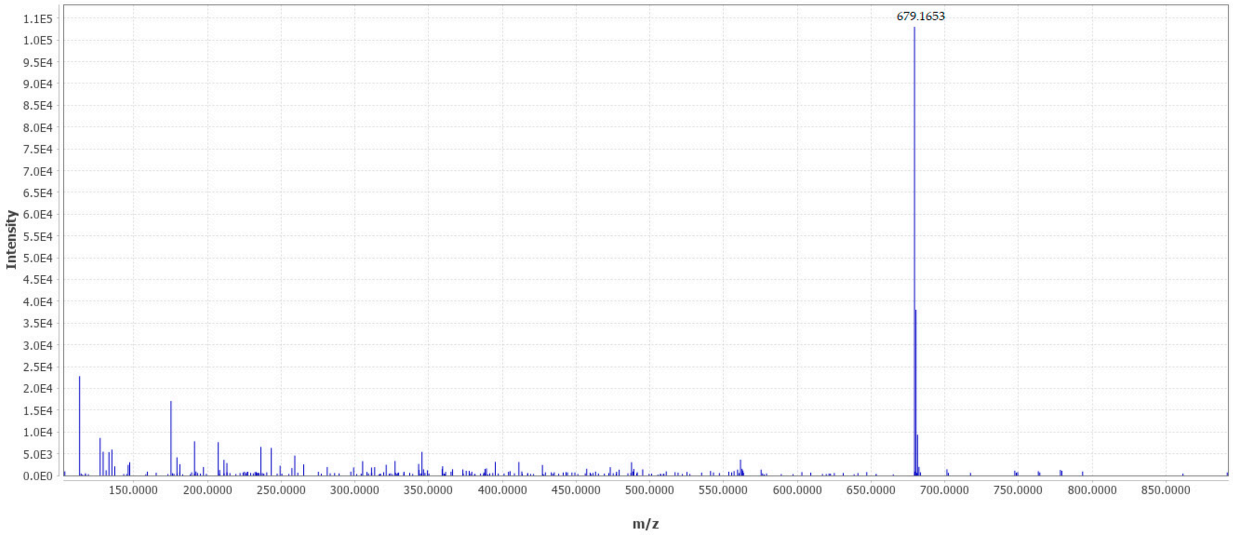

Figure S11. Mass spectrum of peak 11

Table S1. Synergy test of *B. pilosa* extract/Peptide combinations against *C. albicans* SC5314.

Strain	Phenotype	Extract	Peptide	MIC_E	MIC_P	E	P	FIC Index	MIC_E/E	MIC_P/P
C. albicans SC5314	S	8.12	R-1-R	1000	100	250	25	0,50	4	4
			[3A]-R-1-R	1000	100	62,5	50	0,56	16	2
			[2A]-R-1-R	1000	>200	62,5	50	0,31	16	4
			RR-1-R	1000	100	62,5	25	0,31	16	4
			[5Bpa]-R-1-R	1000	>200	62,5	50	0,31	16	4

MIC_E and MIC_P correspond to the MIC (μg/mL) of the extract of *B. pilosa* and peptides derived from R-1-R, respectively; E and P correspond to the MIC values when combining the extract and peptide. Minimum fractional concentration index (FIC I), MIC_E/E, and MIC_P/P represent the factor by which the extract or peptide are potentiated after being evaluated in combination, respectively.

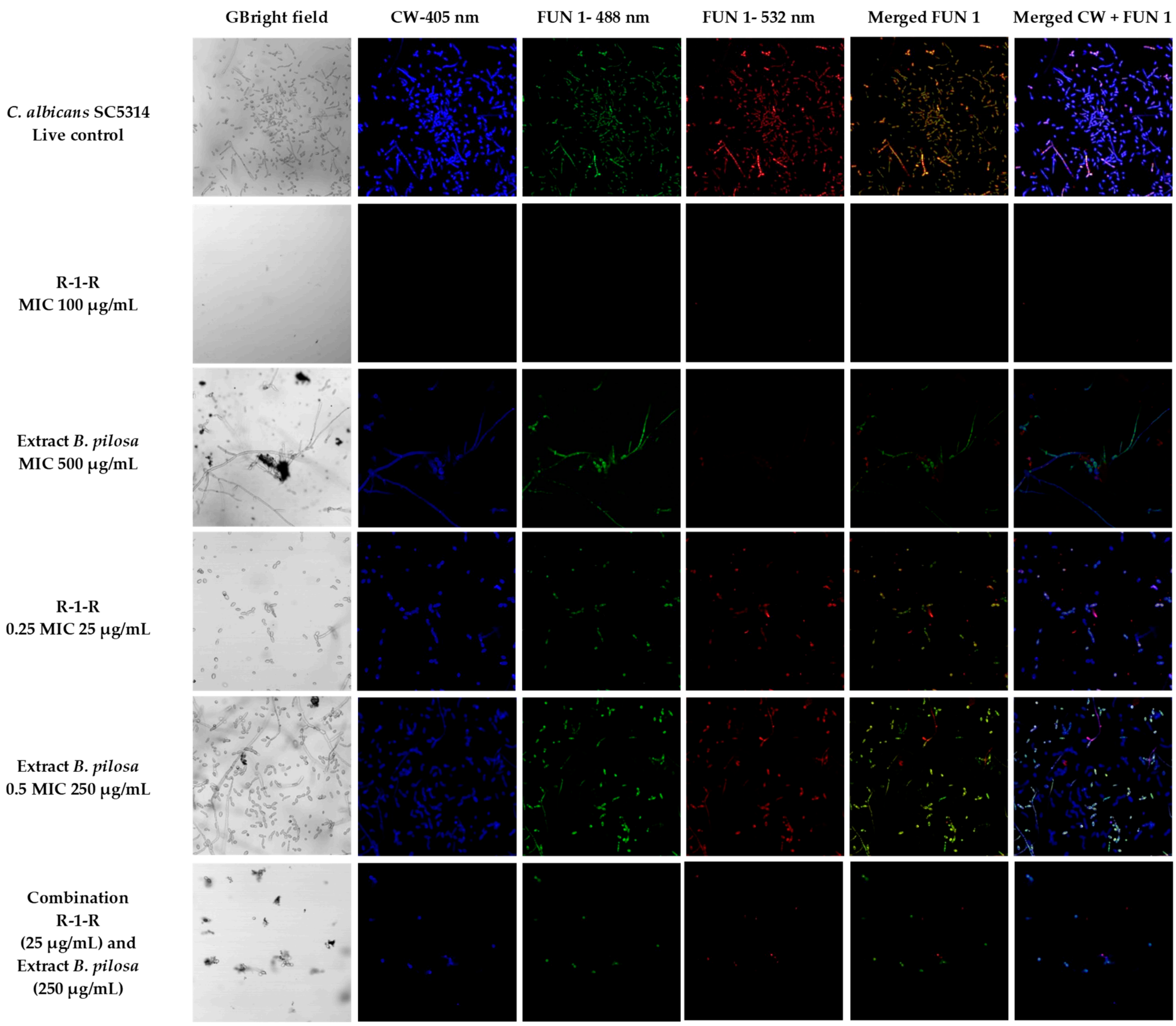

Figure S12. Cell viability assay of *C. albicans* SC5314 after treatment (48 h) with R-1-R, *B. pilosa* extract to MIC value, sub MIC values and combinations of peptide and extract where there was a synergistic effect.

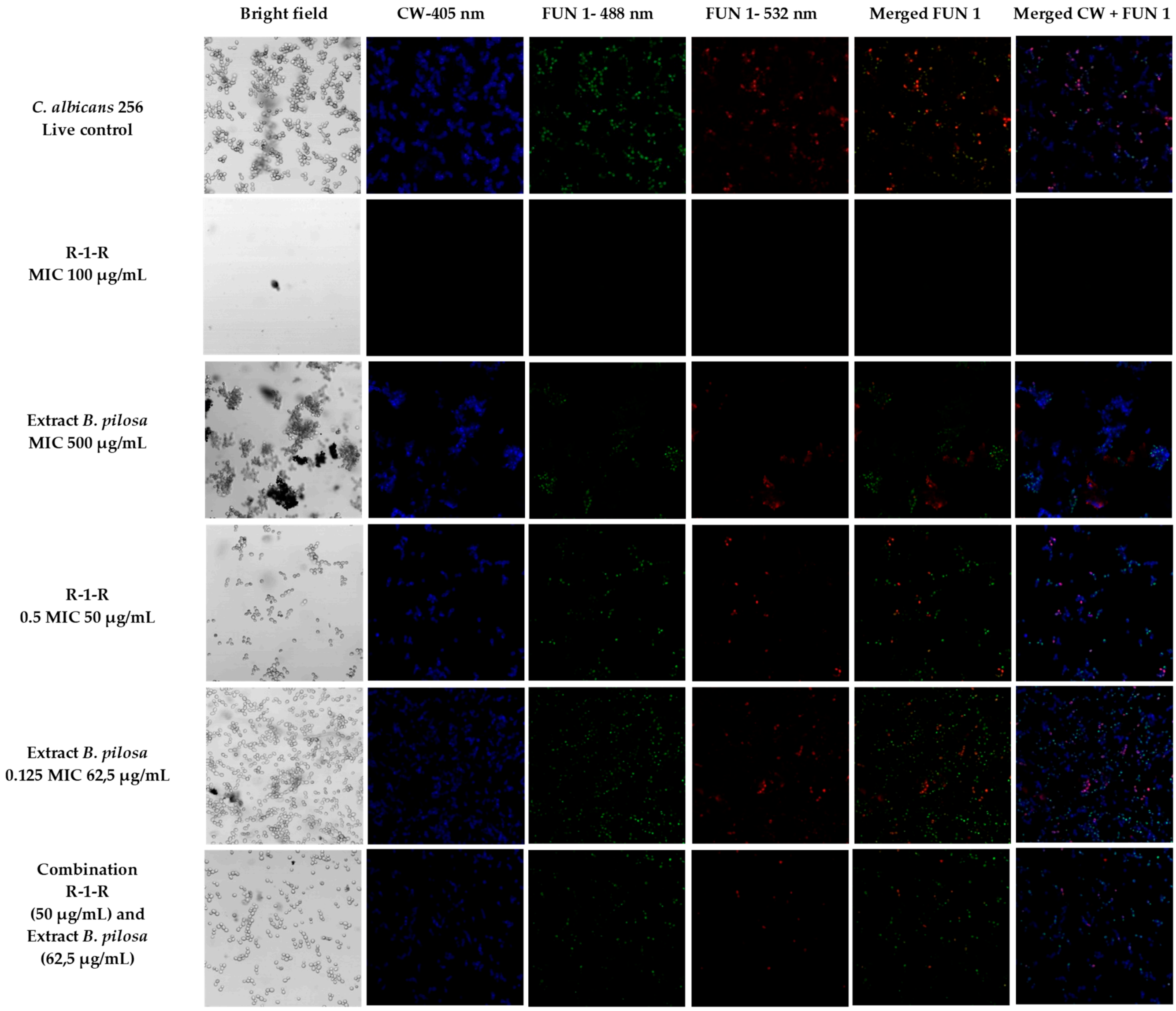

Figure S13. Cell viability assay of *C. albicans* 256 after treatment (48 h) with R-1-R, *B. pilosa* extract to MIC value, sub MIC values and combinations of peptide and extract where there was an additive effect.

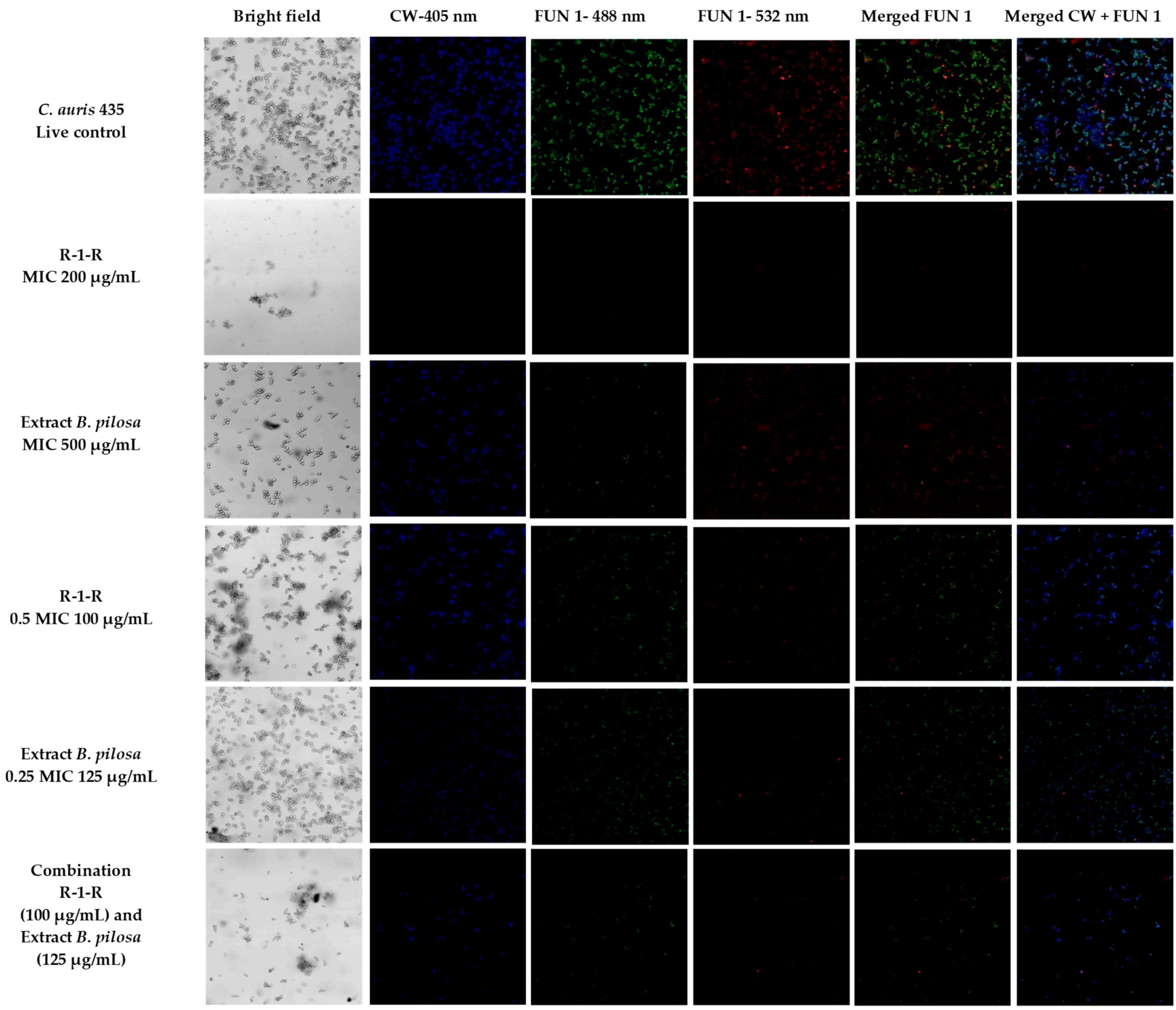

Figure S14. Cell viability assay of *C. auris* 435 after treatment (48 h) with R-1-R, *B. pilosa* extract to MIC value, sub MIC values and combinations of peptide and extract where there was a synergistic effect.

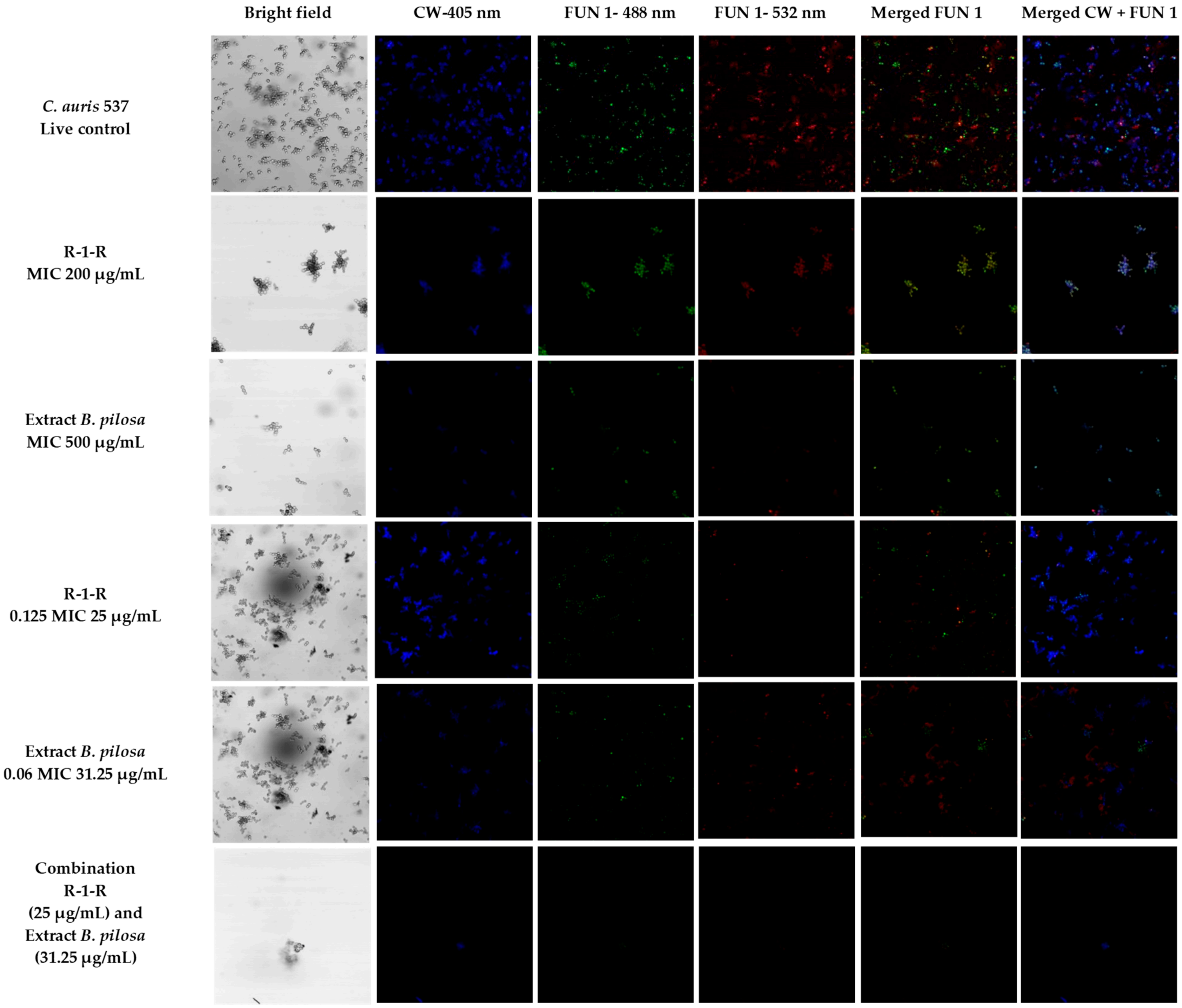

Figure S15. Cell viability assay of *C. auris* 537 after treatment (48 h) with R-1-R, *B. pilosa* extract to MIC value, sub MIC values and combinations of peptide and extract where there was a synergistic effect.
